# Binding Capabilities of Different Genetically Engineered pVIII Proteins of the Filamentous M13/Fd Virus and Single-Walled Carbon Nanotubes

**DOI:** 10.3390/nano12030398

**Published:** 2022-01-26

**Authors:** Amro Sweedan, Yachin Cohen, Sima Yaron, Muhammad Y. Bashouti

**Affiliations:** 1 The Ilse-Katz Institute for Nanoscale Science & Technology, Ben-Gurion University of the Negev, Beer-Sheva 8410501, Israel; sweedan@post.bgu.ac.il; 2 The Russell Berrie Nanotechnology Institute, Technion-Israel Institute of Technology, Haifa 3200003, Israel; 3 Department of Chemical Engineering, Technion-Israel Institute of Technology, Haifa 3200003, Israel; 4 Department of Biotechnology and Food Engineering, Technion-Israel Institute of Technology, Haifa 3200003, Israel; 5 Jacob Blaustein Institutes for Desert Research, Sede Boqer Campus, Ben-Gurion University of the Negev, Sede Boqer 8499000, Israel

**Keywords:** SWNTs, filamentous phage, recombinant virus

## Abstract

Binding functional biomolecules to non-biological materials, such as single-walled carbon nanotubes (SWNTs), is a challenging task with relevance for different applications. However, no one has yet undertaken a comparison of the binding of SWNTs to different recombinant filamentous viruses (phages) bioengineered to contain different binding peptides fused to the virus coat proteins. This is important due to the range of possible binding efficiencies and scenarios that may arise when the protein’s amino acid sequence is modified, since the peptides may alter the virus’s biological properties or they may behave differently when they are in the context of being displayed on the virus coat protein; in addition, non-engineered viruses may non-specifically adsorb to SWNTs. To test these possibilities, we used four recombinant phage templates and the wild type. In the first circumstance, we observed different binding capabilities and biological functional alterations; e.g., some peptides, in the context of viral templates, did not bind to SWNTs, although it was proven that the bare peptide did. The second circumstance was excluded, as the wild-type virus was found to hardly bind to the SWNTs. These results may be relevant to the possible use of the virus as a “SWNT shuttle” in nano-scale self-assembly, particularly since the pIII proteins are free to act as binding-directing agents. Therefore, knowledge of the differences between and efficiencies of SWNT binding templates may help in choosing better binding phages or peptides for possible future applications and industrial mass production.

## 1. Introduction

**Figure 1:**
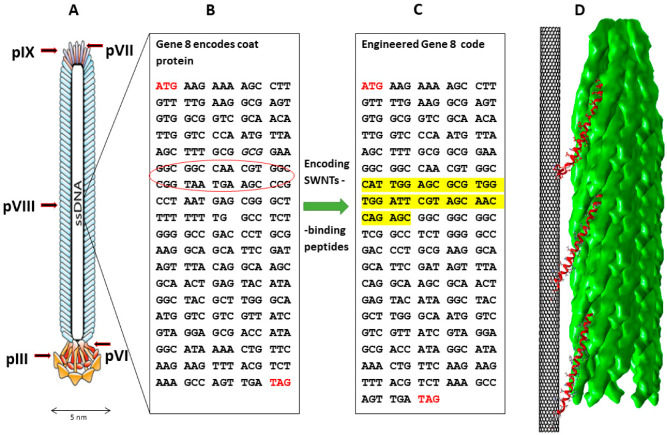
Schematic illustration of the Fd filamentous virus structure, genome engineering, and SWNT binding. (**A**) Schematic model showing the virus ssDNA and five different coat proteins. The ssDNA is in black, pIII is in yellow, and the pVIII coat protein is in blue [52]. (**B**) Native code of gVIII encoding the native virus’s major coat protein; the red circle highlights the inserted engineering region. (**C**) Engineered gVIII code formed by encoding the exogenous (highlighted in yellow) code for SWNT-binding peptides isolated from a phage display library. (**D**) Three-dimensional structural model demonstrating the binding of SWNTs by the Fd virus filament through the fused pVIII–SWNT binding peptides (red). The model is based on the coordinations of the coat protein in fd filamentous bacteriophage particles determined by solid-state NMR spectroscopy [53]. Molecular graphics and analyses performed with UCSF Chimera software [54].

Single-walled carbon nanotubes are exciting nanomaterials because of their unique physical and chemical properties. They are widely deployed for different applications in materials science, nanotechnology, sensor technology, and electronics and are also used for several biological and biomedical applications [1,2,3,4,5,6,7,8,9]. As a result of their properties, proofs of concept for several high-performance devices already exist. However, many serious issues remain to be addressed before the scale-up of these applications can be realized and their viability verified [10]. Specifically, truly innovative applications of these materials will arise from the utilization of surface modifications, handling techniques, deposition methods, and coating technologies, such as (bio)chemically directed self-assembly to enable efficient combinations of several building blocks in functional devices [11,12,13] and (non)covalent molecular modifications that can provide additional benefits [14]. Recently, the interaction of functional biological molecules with SWNTs, which provide them with specific chemical handles, has been reported by different research groups [15,16,17,18,19,20], including polypeptides with selectivity for binding to SWNTs derived from a directed evolution combinatorial library [21,22,23,24,25,26,27,28,29]. The M13/Fd virus, a filamentous bacteriophage, can be genetically engineered to display functional biological molecules (peptides) selected for binding to non-biological materials (such as SWNTs) [30,31,32]. The wild-type bacteriophage is composed of circular single-stranded DNA (ssDNA), 6407 nucleotides long and encapsulated in a tube made of approximately 2700 copies of the major coat protein (pVIII), capped with four different minor coat proteins (pVII, pIX, pIII, and pVI) on the ends, as demonstrated in Figure 1A [30,31,32]. The phage can infect *Escherichia coli* bacteria (*E. coli*). Utilization of the M13 template as a programmable method to integrate SWNTs into photovoltaic and electronic devices [33,34], imaging probes [35,36,37], and conductive nanomesh [38,39,40,41,42] has been reported. However, to the best of our knowledge, no one has yet demonstrated solid evidence of the binding efficiency of SWNTs to recombinant viruses displaying different binding peptides or to non-modified virus templates. In addition, previous research has not investigated various aspects of this topic, including: (i) determining appropriate controls, (ii) comparing the different binding capabilities of different isolated SWNT-binding peptides, (iii) proving peptide binding in the context of the virus’s major coat protein instead of the bare peptide–SWNT polydispersity, (iv) testing for alterations in virus biological functionality that would be relevant to future mass production, (v) non-specific heteroaggregation between SWNTs and the virus [43], and (vi) excluding wild-type virus contamination. In this article, we propose the use of surfactant-free methods. Surfactants used to prevent SWNT bundling may lead to either a deterioration in the electronic properties of the SWNTs [44] or disruption of different chemical reactions, resulting in more impurities [34]. In addition, binding to SWNTs by peptides, in the context of the phage coat protein, depends on the peptide as a functional molecule, in the context of the phage for suspending SWNTs, more than on the amino acids. This may avoid the protein-unfolding issues observed with other strategies [45].

We report herein a comparison of the binding capabilities of different engineered SWNT-specific viruses (named V4, V19, V23, and V28). The viruses were bioengineered to display nanotube-binding peptides fused to the major coat protein of the virus (pVIII). Our purpose was to bind the SWNTs along the viral filament; hence, we used pVIII rather than pIII/pIX/pVI/pVII. The investigation included precise controls by comparing the binding efficiencies of different modified viruses to the non-engineered wild-type virus and to a virus-free sample. Briefly, this was done by (i) comparing four selected strong SWNT-binding peptides (named P4, P19, P23, and P28) [23,24,25,46] fused to the filamentous virus’s major coat protein (pVIII), (ii) using a surfactant-free procedure (to eliminate the mentioned side effects), (iii) comparing the biological functionality of engineered viruses, such as infection, viral production, and packaging, with that of the wild-type virus, (iv) verifying the precise DNA code sequencing for each modified clone, (v) verifying that the modified peptides were displayed on the engineered virus facing the outside solution and that they were capable of binding in the context of the virus filament, which could differ from the bare peptide in the solution. The results showed that (i) the wild-type virus did not non-specifically bind to SWNTs in the dispersion, (ii) the engineered V4 and V28 viruses successfully bound to SWNTs in the dispersion in the context of the pVIII virus coat protein, (iii) the V23 virus displayed on the phage barely bound to SWNTs in the dispersion despite the successful binding of synthesized bare p23 peptides to SWNTs in the solution [24], (iv) V28 successfully bound to SWNTs in the contexts of pVIII and pIII [46], (v) V19 did not bind to SWNTs in the dispersion, (vi) the infectivity of phages that displayed V4 and V28 was dramatically altered compared to the wild-type phage, (vii) a viral reproduction decline was noticed in V28, V23, and V4 but not in V19, and (viii) a sonication procedure could replace the traditional surfactant methods for surfactant-free SWNT–virus dispersibility. The different binding efficiencies and bio-alterations of the peptides can be attributed to the different physical and chemical properties (e.g., hydrophobicity and π–π interaction) of the displayed recombinant peptide. These physical and chemical properties are directly derived from the peptide’s specific amino acid sequence. This research may be relevant to the possible use of the virus as a “SWNT shuttle” in nano-scale self-assembly, particularly since the pIII proteins are free to act as binding-directing agents [46,47,48,49]. Knowing the differences between and capabilities of a range of SWNT-binding templates should help in choosing better binding viruses or peptides for future applications and mass industrial production. 

## 2. Results and Discussion 

### 2.1. Selecting SWNT-Binding Candidates 

To bioengineer the virus’s major coat (pVIII) protein to display nanotube-binding peptides, four different SWNT-binding peptides, previously isolated by other groups from combinatorial phage display pIII-peptide libraries [23,24,25,46], were selected (P4, P19, P23, and P28; see Table S1) and encoded at the virus’s DNA level. They were selected based on their successful SWNT binding due to their: (i) maximal binding affinity, (ii) SWNT-binding selectivity, (iii) high occurrence in bio-panning cycles, and (iv) dominance as correlated with their binding affinity in bio-panning cycles. In light of these properties, these peptides have been widely used for different applications. For example, the P23 peptide has been used in different SWNT-binding applications, including the functionalization of SWNT field effect transistors [16], the non-covalent control of the solubility of SWNTs [18], and the mediation of the formation of SWNT composites [24]. In addition, bare P23 synthesized peptides can disperse SWNTs in water through sonication to form a homogeneous stable suspension [24]. P28 has been used in fabricating lithium-ion battery electrodes [46]. These four peptides are encoded at the DNA code level (Table S2), as depicted in Figure 1. Additional details on bioengineering approaches, such as why certain residues are removed/left [50], why new residues are added, and details pertaining to the ‘type 88’ virus system [51], can be found in the Supplementary Material. 

### 2.2. Engineered Virus Quantification 

The wild-type and the four engineered viruses—V4, V19, V23, and V28, displaying P4, P19, P23, and P28, respectively (Table S1)—were successfully purified and titered to determine the viruses’ concentrations (Table 1).

Titers were recorded as the averages of three different virus purifications: 6 × 10^10^ mL^−1^, 1.2 × 10^12^ mL^−1^, 1.5 × 10^12^ mL^−1^, and 5 × 10^11^ mL^−1^ for V4, V19, V23, and V28, respectively. However, the foreign insert that was intended to be displayed via engineered phage proteins can have a negative effect on protein expression, phage assembly, and infectivity. Table 1 shows the engineered virus titers compared to the non-modified virus, and the results imply infection alteration or assembly disruption of V4 and V28. Biological titer determination is based only on the viral infection of susceptible bacteria; thus, we do not know, at this stage, whether the results were due to a lower viral concentration in the sample or infection disruption (viruses were produced and secreted in solution but did not infect the bacteria). Lower concentrations were observed with the V4 and V28 titers, which were roughly 5–20-fold lower (1:21 and 1:2.5, respectively) than the native titer. To determine the true viral particle concentration (which is not correlated with viral infection), we measured the relative concentration of the virus clones with Coomassie brilliant blue total protein quantification (see the Methods section). As shown in Table 1, V4 and V28 exhibited large differences in viral titer compared to the native virus, whereas the relative concentration decreased to a similar fold after using the relative protein concentration. We can, therefore, conclude that the different results in the quantification seem to be due to viral infection alteration and not to viral assembly or secretion (i.e., viral production). Compared to the biological titer, total protein quantification is generally more convenient, as well as being more tolerant to virus structural alteration. Knowing the differences between different SWNT-binding viruses can help in choosing better binding viruses for future applications and mass industrial production. 

### 2.3. SWNT-Binding Peptides Display Verification 

**Figure 2:**
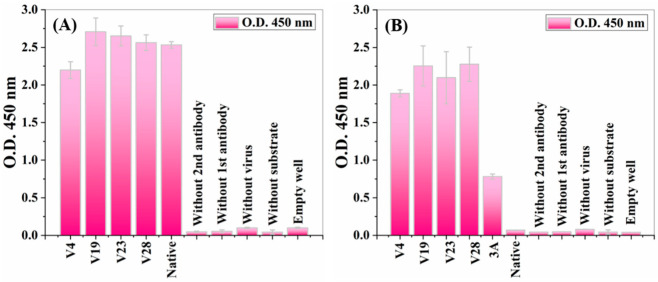
Virus (phage) ELISA for determining the presence of recombinant pVIII in the recombinant virus structure. ELISA wells, coated with mouse anti-Fd (anti-M13 phage) antibodies, were used to capture six different phages: four chimeric phages displaying SWNT-binding peptides on protein VIII and containing both the recombinant and the wild-type protein pVIII (V4, V19, V23, V28); a recombinant virus displaying a peptide library as a true positive control (3A); and a wild-type native virus. The captured phages were reacted with a rabbit anti-recombinant pVIII antibody and rabbit anti-Fd phage polyclonal antibodies. Bars represent standard deviations of duplicate measurements from a single experiment. (**A**) Anti-Fd general antibody ELISA against four recombinant SWNT-binding peptides displaying viruses (V4, V19, V23, V28) and a native virus. (**B**) Anti-recombinant pVIII antibody ELISA against the same clones from the previously mentioned ELISA and a 3A clone. The 3A clone was a recombinant phage displaying a peptide library (a true positive control). Several false-positive controls were included on each ELISA: wells without a primary/secondary antibody, wells without viruses/substrates, and an empty well.

Engineered virus clones with corresponding SWNT-binding peptides were verified and tested. To detect the incorporated engineered pVIII in the virus’s cylindrical structure, ELISA, with a specific anti-recombinant pVIII protein antibody, was used and an anti-M13 virus general antibody ELISA was included as a control. A general antibody is a critical control to verify a virus’s existence in samples. The phages were captured on the surface of the ELISA plate, which was coated with general mouse anti-M13 phage antibodies. The primary detection antibodies were either general rabbit anti-M13 phage antibodies or rabbit anti-recombinant pVIII antibodies. The horseradish peroxidase-conjugated anti-IgG secondary anti-bodies were anti-rabbit antibodies for prevention of cross-reactivity toward mouse coating/capturing general anti-M13 phage antibodies. Based on the relative total protein concentration (Table 1), two virus samples (V19 and the non-modified virus) were diluted to obtain similar relative protein concentrations (V4, V23, and V28 were undiluted). Coomassie brilliant blue quantifications for the diluted samples are shown in Figure S1. The ELISA results for the virus samples are shown in Figure 2.

**Figure 3:**
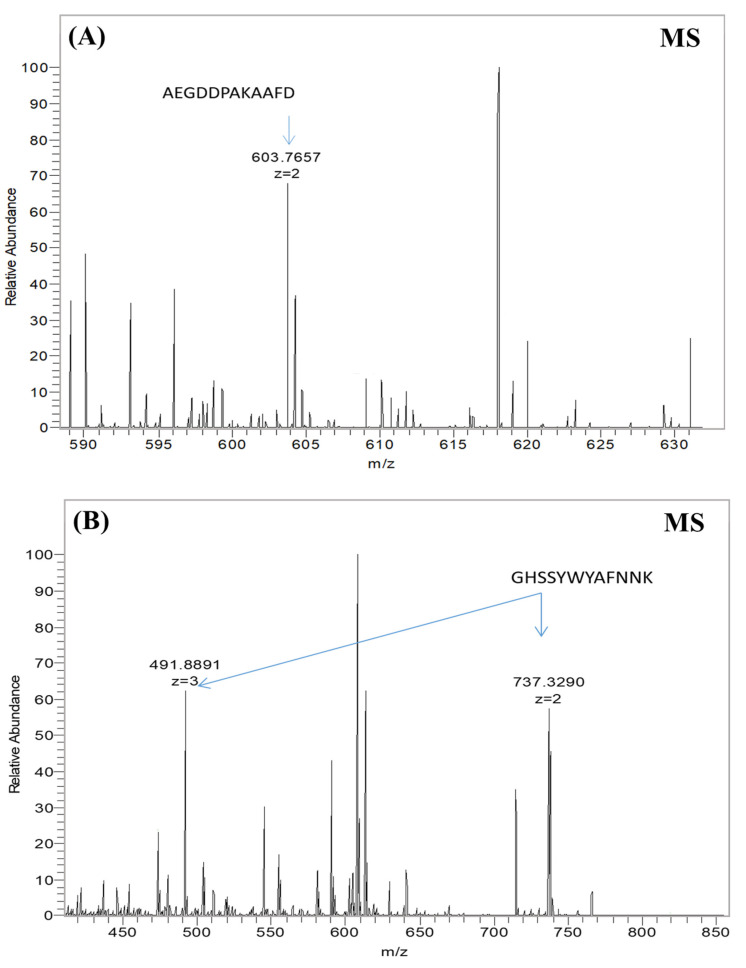
Mass spectra data for the native virus and the V23 modified virus. Bars represent ionized peptide fragments with a specific mass-to-charge ratio (*m/z*); the length of the bar indicates the relative abundance of the ion. (**A**) Native virus sample, with the relevant peak of the native coat protein sequence indicated by an arrow. (**B**) V23 virus sample, in which a recombinant P23–pVIII protein sequence was detected; the two relevant peaks of the engineered pVIII are indicated by arrows.

At a similar optical signal level, the anti-M13 general antibody (referred to as anti-Fd) was supposed to recognize and bind to both engineered and native non-modified viruses. These ELISA results illustrated that general anti-M13 antibodies efficiently bind to both native and engineered phages (Figure 2A). Similar levels of binding signals illustrated equal overall phage levels for all five phage types (four engineered and one native). These results indicated that all five phage types were present in equal amounts, supporting the Coomassie brilliant blue quantification (Figure S1). An anti-recombinant pVIII antibody [55] was tested to check the existence and accessibility from the solution of the modified pVIII coat proteins on the virus’s surface. An anti-rec pVIII antibody was tested for binding against six virus clones: the wild-type native virus, four types of engineered viruses displaying the SWNT-binding peptide (V4, V19, V23, V28), and a recombinant virus displaying a peptide library fused to pVIII (A3) as a true positive control (Figure 2B). Signals obtained from the anti-rec pVIII antibody ELISA showed that the antibodies efficiently bound only to the engineered viruses (V4, V19, V23, V28) and the 3A true positive control, whereas the native non-modified viruses showed no binding signal. The lower optical density (O.D.) for the positive 3A control compared to the virus samples was due to the lower display concentration of the bioengineered proteins in the virus structure. These binding signals indicated that all the engineered viruses contained recombinant pVIII copies incorporated into each phage structure, unlike the native viruses. Moreover, the engineered SWNT-binding peptides were accessible and were not buried in the viral structure (there was no steric hindrance). In addition, mass spectroscopy provided another method of verifying each SWNT-binding peptide at the protein sequence level and of determining the composition of each recombinant phage sample (Figure 3B). The mass spectrum of the native virus had a single peak at 603.7 (*m*/*z* = 603.7 amu), corresponding to the mass-to-charge ratio of an amino acid fragment due to the native pVIII proteins (Figure 3A). The two additional peaks at 491.8 and 737.3 (*m*/*z* = 491.8/737.3 amu) corresponded to the mass-to-charge ratio of the engineered p23–pVIII proteins, indicating that the SWNT-binding peptides were displayed on the pVIII proteins of the bacteriophage mosaic. Similar experiments were undertaken for V19, V4, and V28 (data not shown).

### 2.4. Virus Thermal and Sonication Stability

**Figure 4:**
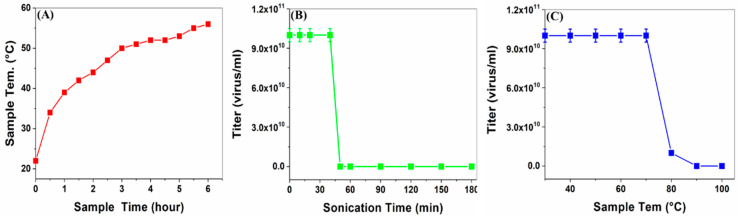
Determination of virus’s thermal and sonication stability. (**A**) Sample temperature change during sonication. (**B**) Virus viability correlation with sonication time. (**C**) Virus viability correlation with sample’s increasing temperature.

Before preparing SWNT–virus samples, the virus’s thermal and sonication stabilities were assayed. The stability threshold was determined by testing the virus’s biological titer drop (Figure 4). The findings indicated that the virus was stable up to ~70 °C (Figure 4C) and after exposure for ~40 min in a sonication bath (Figure 4B). These safety intervals were considered in the following experiments. Moreover, increasing the sample temperature during sonication indicated that the samples were within the safe temperature interval for >5 h (Figure 4A).

### 2.5. SWNT–Virus Binding Characterization 

**Figure 5:**
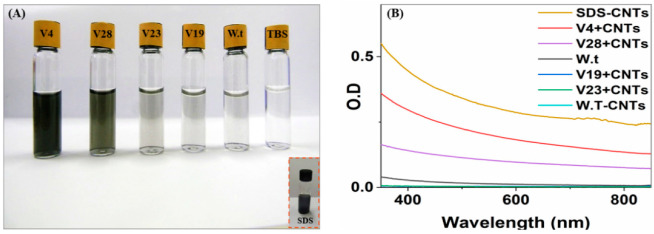
Virus–SWNT sonication results and spectrophotometric wave-scans. (**A**) Photographs of virus–SWNT vials after sonication and filtration, showing the SWNT suspension with four recombinant virus clones, the native virus, and the buffer; the SDS detergent suspension is also depicted for comparison. Differences between the peptide/SWNT dispersions were visually detected from the disparity in supernatant color intensity, which qualitatively indicated the quantity of dispersed SWNTs. (**B**) Spectrophotometric wave-scan of four engineered clones with SWNTs and of SDS–SWNTs.

Based on all these observations, we prepared SWNT–virus samples (V4, V19, V23, and V28) in Tris-buffered saline (TBS buffer). Sono-chemistry protocols were used to disperse the SWNT bundles without using surfactants (Figure 5). The full details for the sample preparation are presented in the Methods section. Based solely on the supernatant’s color intensity, the quantity of SWNTs solubilized appeared to be much higher in the V4 and V28 virus samples than in the native non-modified virus. Compared to a control experiment, the results for the latter two dispersions were similar to those for the dispersion obtained with SDS–SWNTs, providing evidence that V4 and V28 are capable of binding and dispersing SWNTs in dispersions. The V19 and V23 solutions were nearly transparent, similar to the non-modified virus solution, implying poor SWNT binding and dispersion. In general, the virus-binding efficiency depends on two major factors: the number of incorporated copies of the SWNT-binding peptide (avidity factor) and the specific SWNT-binding affinity of each peptide in the context of the virus coat protein (affinity factor) [56]. The relative binding efficiency of each modified virus in suspension can be calculated through a comparison to the non-modified virus SWNTs’ resuspension ability. The calculation is based on the quantity of successfully resuspended SWNTs in the TBS buffer visualized with UVIS spectroscopy. Starting with a similar virus concentration for each sample, V4, V28, V23, and V19 increased their binding of SWNTs by almost 290-, 140-, 5-, and 4-fold, respectively (Figure 5B), compared to the non-modified virus. Comparing V4 and V28, the difference in binding efficiency seems to have been due to a higher peptide–SWNT binding affinity. V28 appeared to have more binding-peptide copies in its coat protein (Figure 2B). This means that the improved binding efficiency was due to the higher affinity of V4 peptide–SWNTs. V19 and V23 bound SWNTs in a similar manner to non-modified viruses. The poor binding of V19 and V23 can be attributed to several factors. (i) The combinatorial peptide display libraries used were ‘type 3’ fusion constructs; thus, the binding peptides isolated were in the context of the pIII rather than the pVIII coat protein. The pIII protein is much longer than pVIII and has less steric disruption, which might have interfered with SWNT binding in dispersions. Our purpose was to bind the SWNTs along the viral filament; hence, we used pVIII rather than pIII. (ii) Our 3D theoretical ribbon models (see Figure S2) of the P4, P19, P23, and P28 peptides generated by the PEP-FOLD server [57] indicated a more compact folded structure for P19 and P23 compared to the open and coiled P4 and P28, thereby supporting our hypothesis of steric disruption interference. (iii) The binding of hydrophobic amino acids was not possible due to hydrophobic intermolecular or intramolecular interactions. Future analysis of the effects of the peptides and SWNTs on the structures of the viruses would help elucidate the factors that affect the interaction between peptides and SWNTs in the context of the peptide phage display system.

**Figure 6:**
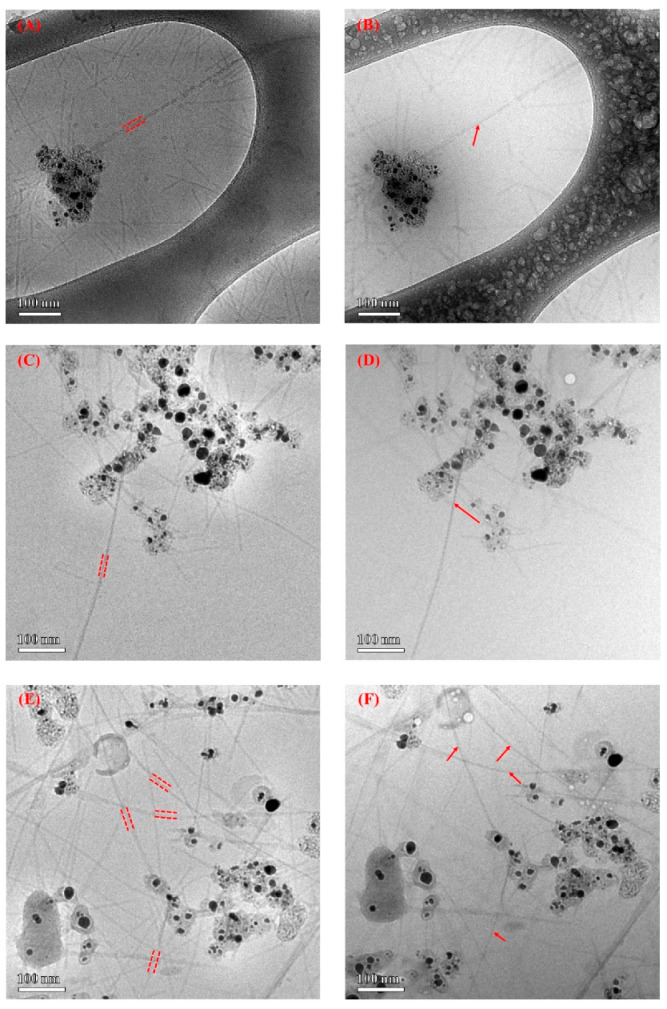

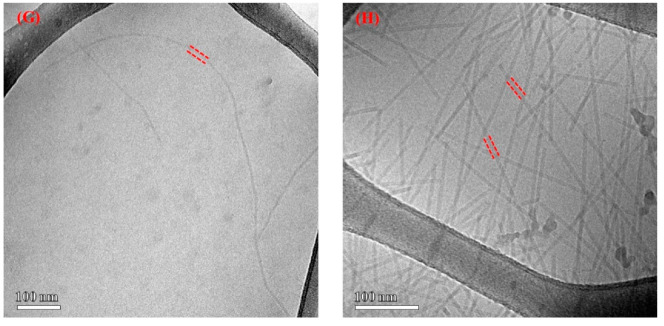
Cryo-TEM images of SWNT–virus samples. (**A**–**F**) are TEM images of different SWNT–V28 samples before and after irradiation, respectively. The SWNT is indicated by an arrow and the virus is indicated by dashed lines. Part of the virus was intentionally burned off during imaging (radiolysis) to more clearly identify the bound SWNTs. The black extraneous material is residual catalyst coated with amorphous carbon. (**G**) shows the wild-type native virus dispersion without absorbed SWNTs. (**H**) is a TEM image of SWNT–V28. The native virus without SWNTs seemed quite wiggly and appeared to be stiff after SWNT adsorption along the engineered virus in (**A**–**F**).

Successfully bound virus samples were visualized using cryo-transmission electron microscopy (cryo-TEM) to characterize bound SWNTs along the major coat proteins of the virus filament (Figure 6). The TEM images showed that the engineered virus was capable of binding to the SWNTs and could remain stable even after sonication. TEM electron radiolysis during imaging identified the bound SWNTs along the viral filament after biomolecule decomposition. 

## 3. Conclusions and Summary

Four virus clones displaying P4, P19, P23, and P28 SWNT-binding peptides were produced. The peptides were selected based on their SWNT-binding affinity and their reported reliability. The four designed Fd viruses with SWNT-binding peptides underwent successful assembly and proved to be functional and viable. Virus cultural purification results showed similar relative concentrations for each engineered virus sample (V4, V19, V23, and V28) compared to the native non-engineered Fd. The biological titers (concentration was based on virus infection) of V4 and V28 were roughly 5–20-fold lower than the native virus. The differences between the concentrations obtained by Coomassie brilliant blue quantification and the virus biological titer suggest a negative effect on the biological infection of V4 and V28, which can be attributed to a structural modification of the coat proteins. On the other hand, the assembly and secretion of the four engineered viruses seemed to be unaltered. Modified phage genome fragment sequencing proved that each clone genotypically contained the appropriate DNA encoding SWNT-binding peptide sequences. Phenotypic expression, corresponding to the SWNT-binding peptides displayed on the virus filament, was verified through a specific anti-recombinant pVIII (rec pVIII) antibody ELISA and mass spectrometry. The ELISA binding data indicated that the SWNT-binding peptides were displayed, that they contained the correct amino acid sequences, and that they were accessible to the solution molecules. Visual inspection of the sonicated dispersions of the modified virus/SWNTs and the non-modified virus/SWNTs highlighted differences in the dispersion abilities since the color intensity indicated the quantity of dispersed SWNTs. The binding efficiency of the viruses to the SWNTs strongly depended on the peptide used. The quantity of dispersed SWNTs seemed to be higher in the V4 and V28 virus samples than in the native non-modified virus. Cryo-TEM visualization showed bound SWNTs along the major coat proteins of the virus filament. This may be relevant to the possible use of the virus as a “shuttle,” particularly since the pIII, pIV, pXI, and pVII proteins are free to act as binding-directing agents. The different binding capabilities of the different peptides are correlated with each peptide’s physical and chemical properties, derived directly from the peptide’s specific amino acid sequence, as previously mentioned. The engineered M13 virus may pave the way for using aligned and highly organized SWNT–virus samples as anisotropic molecular building blocks for nanoscale devices and circuits. Further studies can expand this system by adding compatible functionalization and selectivity to the rational design section. A significant advantage of using biological M13 system peptides to bind nanotubes is that the process can be controlled by specifying the amino acid sequence at the DNA level. We exploited this advantage in this study to engineer a self-binding function into the peptides, but other capabilities can also be envisioned. Other than pVIII engineering, the virus genome bio-system enables further genome modifications that could be applied in the future to produce other specific modifications in defined regions of the virus filaments; for example, biotinylating on specific amino acids assembled in the virus’s cylindrical structure or displaying semiconductor or metal-binding peptides as linkers in specific positions and orientations, producing an ordered hybrid biostructure. Finally, this work might be a step towards arranging SWNTs into architectures that are useful for electrical circuits and molecular-sensing applications. As such, specific modifications of the pIII region with given functionalities, such as an affinity to a specific semiconductor or metal nanoparticle, solid face, or targeting agent (e.g., using the biotin/streptavidin system), could allow the virus to serve as a shuttle for connecting SWNTs to these systems.

## 4. Methods

Materials. The Fth1 phage vector was kindly provided by Prof. Jonathan Gershoni (Tel Aviv University, Tel Aviv, Israel) [58]. The virus genetic code has been deposited in GenBank as accession no. AF362081 [59]. *Sfi*I restriction enzymes were purchased from New England Biolabs (NEB). Oligonucleotides and PCR primers were from Sigma-Aldrich. DH5α *Escherichia coli* bacteria were obtained from Prof. Jonathan Gershoni (Tel Aviv University, Tel Aviv, Israel) [58]. DNA polymerase and dNTP were purchased from Bioline. The DNA ligase was purchased from Promega, tetracycline from Sigma-Aldrich, the JETstar Plasmid Maxiprep kit was obtained from GENOMED GmbH, the Bio-Rad protein assay kit was purchased from Bio-Rad, and the SWNT powders were obtained from CarboLex; they were produced by arc-discharge technology and contained about 30 wt. % carbon impurities and nickel/yttrium catalyst particles. The anti-Fd bacteriophage rabbit IgG primate antibody was from Sigma-Aldrich. The anti-recombinant pVIII primary antibody was kindly provided by Prof. Jonathan Gershoni (Tel Aviv University, Tel Aviv, Israel) [55]. We used SWNTs as a representative for all types of CNTs. The (HRP-)conjugated anti-IgG secondary antibodies were purchased from Jackson ImmunoResearch, and the Coomassie brilliant blue reagent was obtained from Bio-Rad. All buffers were prepared in-house as described [60]. CaCl_2_, MnCl_2_, MgCl_2_, NaCl, HCl, and KCl were purchased from Merck. Acetic acid, PEG 800, glycerol, and Tris were obtained from Sigma-Aldrich. Bacterial media were purchased from Difco. ‘type 88’ virus design: All protocols were as previously described [60] unless otherwise noted. The Fth1 phage vector was propagated in a DH5α *E. coli* overnight culture before the phage DNA was isolated with a JETstar Plasmid Maxiprep kit according to the manufacturer’s instructions. The isolated vector DNA was verified by fragment sequencing, the DNA 1% gel electrophoresis pattern, and restriction site mapping (data not shown). The Fth1 phage vector pVIII cloning site was digested with the *Sfi*I restriction enzyme according to the manufacturer’s instructions. SWNT-binding peptides were encoded at the DNA level according to *E. coli* codon usage [61]; four DNA sequences (single-stranded oligonucleotides with overlapping regions), named N4, N19, N23, and N28 (see Table S2), were annealed to generate duplexes by heating them to 90 °C for 5 min and allowing them to cool at room temperature for 1 h. Each double-stranded DNA duplex was separately cloned to a digested Fth1 vector before vector inserts were ligated by T4 DNA ligase following the manufacturer’s instructions. The ligated vectors were transfected to DH5α *E. coli*-competent cells by heat shock and plated on tetracycline agar plates. Colonies that contained the four phage inserts were propagated to produce the engineered viruses. The four modified viruses (V4, V19, V23, V28) were purified and titered. For the sake of clarification, we named the peptides after their sequences (for example, P4 was named after N4). Determination of viral particle concentration: The virus particle titer was measured by infecting DH5α *E. coli* bacteria with serial dilutions of the phages, plating and incubating the tetracycline agar plates overnight at 37 °C. On the following day, the titer was calculated by counting the tetracycline-resistant bacterial colonies and multiplying the number by the dilution factor. The relative quantity of physical virus particles was evaluated with the Coomassie brilliant blue protein reagent following the manufacturer’s instructions, an enzyme-linked immunosorbent assay (ELISA), and sample spectrophotometric absorption measurements from 220 nm to 340 nm using a UV-visible spectrophotometer and 1-cm optical-path quartz cuvettes. Virus thermal and sonication stability: Ultrasonication was carried out in a sonicator bath for a predetermined time, with 40 K Hz frequency and 150 watt rated power. The thermal stress test was carried out in a heated bath that contained a digital thermometer with thermostat control. The viral titer was determined with different time intervals in both a sonicator bath and thermal bath. Molecular verification of a viral particle: Engineered DNA fragments of viruses were sequenced for code verification. Biological infectivity and functionality were checked. An anti-recombinant pVIII ELISA for recombinant pVIII detection was used as previously described [55], and mass spectrometry was used for engineered peptide detection. Samples for mass spectrometry were trypsinized, and their tryptic peptides were analyzed by LC-MSMS on an OrbitrapXL mass spectrometer (Thermo). The data were analyzed with Sequest 3.31 software vs. the human section of the Uniprot database. Cryo-TEM characterization: Specimens of SWNT–phages, used for characterization with cryo-TEM, were prepared in a controlled-environment vitrification system (CEVS) at 25 °C and 100% relative humidity. Samples were examined using an FEI T12 G2 transmission electron microscope (Eindhoven, The Netherlands) at 120 kV, with Oxford CT-3500 or Gatan 626 cooling holders and transfer stations. Specimens were equilibrated in the microscope below −178 °C and examined in the low-dose imaging mode to minimize damage due to electron-beam radiation. Images were recorded at a nominal under-focus of 1–2 μm to enhance phase contrast. Images were acquired digitally with a Gatan US1000 high-resolution (T12) cooled CCD camera using Digital Micrograph software. SWNT–virus sample preparation: A 2 mL sample with a 1:10 SWNT-to-phage weight ratio was added to TBS buffer (0.05 M Tris-buffered saline, NaCl 0.138 M; KCl 0.0027 M; pH 8.0) and sonicated for 45 min in a sonicator bath at 40 KHz frequency and 150 watt rated power. The upper 60% of the supernatant, after sonication, was carefully collected and centrifuged at 10,000 g for 1 min before another round of upper-supernatant collection.

## Figures and Tables

**Table 1:** Viruses’ biological titer and Coomassie brilliant blue total protein quantification.

Virus Clone	V4	V19	V23	V28	Native
Titer (viruses/mL)	6 × 10^10^	1.2 × 10^12^	1.5 × 10^12^	5 × 10^11^	1.26 × 10^12^
Titer standard error	0.47	1.16	0.72	1.74	0.52
Relative titer	1	20	25	8.3	21
Relative protein concentration	1	2.3	1.1	1.2	1.9

## Data Availability

Data available within the article or its supplementary materials.

## Supplementary Materials

The following are available online at https://www.mdpi.com/article/10.3390/nano12030398/s1, Figure S1: Diluted virus samples’ quantification. Total virus proteins’ relative concentration for each virus sample. Dilution indicates nearly equal overall phage levels for all five phage types (V4, V19, V23, V28, and native). These results indicate that all five phage types are present in equal amounts with similar relative concentrations of all virus samples; Figure S2: 3D ribbon models of P4, P19, P23, and P28 SWNT-binding peptides. Each peptide N-terminal end exposed to the solution and C-terminal linker ends are indicated. Right and left sides depict each peptide backbone with and without side chain residues, respectively. Colors indicate amino acid hydrophobicity; the red to white gradient indicates the maximal to minimal hydropathy, respectively; Table S1: Sequences of the four peptides used in our study, called P4, P19, P23, and P28; Table S2: Annealed duplexes prepared for cloning. *Sfi*I sites are colored in red, GGGS linkers in green, and SWNT-binding peptide inserts in black.
