# Supplementary material for: Binding Capabilities of Different Genetically Engineered pVIII Proteins of the Filamentous M13/Fd Virus and Single-Walled Carbon Nanotubes
Source: Nanomaterials (Basel). 2022 Jan 26;12(3):398. doi: 10.3390/nano12030398 (PMC8839290; doi:10.3390/nano12030398)
Supplement: Supplementary file 1 [file nanomaterials-12-00398-s001.zip › nanomaterials-1525396-Supplementary Materials/nanomaterials-1525396-Supplementary Materials.pdf]

SUPLEMENTARY MATERIAL

Binding Capabilities of Different Genetically Engineered pVIII Proteins of the Filamentous M13/Fd Virus and Single-Walled Carbon Nanotubes

Amro Sweedan [1,2], Yachin Cohen [2,3,*], Sima Yaron [2,4,*] and Muhammad Y. Bashouti [1,5,*]

1 The Ilse-Katz Institute for Nanoscale Science & Technology, Ben-Gurion University of the Negev, Beer-Sheva 8410501, Israel; sweedan@post.bgu.ac.il
2 The Russell Berrie Nanotechnology Institute, Technion-Israel Institute of Technology, Haifa 3200, Israel
3 Department of Chemical Engineering, Technion-Israel Institute of Technology, Haifa 3200, Israel
4 Department of Biotechnology and Food Engineering, Technion-Israel Institute of Technology, Haifa 3200, Israel
5 Jacob Blaustein Institutes for Desert Research, Sede Boqer Campus, Ben-Gurion University of the Negev, Sede Boqer 8499000, Israel
* Correspondence: yachinc@technion.ac.il (Y.C.); simay@technion.ac.il (S.Y.); bashouti@bgu.ac.il (M.Y.B.)

Information regarding our bioengineering approach:

SWNT-binding peptides, selected from combinatorial libraries, were fused to the Fd virus pVIII coat protein. Modifying pVIII was achieved by encoding each SWNT-binding peptide in the Fd virus's DNA. In order to engineer pVIII fused to SWNT-binding peptides, several steps were taken: 1) SWNT-binding peptides were fused upstream to the pVIII N-terminal end that was exposed to the solution; 2) an open reading frame (ORF) was considered; 3) the AEG (Ala-Glu-Gly) native N-terminal end was left to improve virus solubility; 4) A 'type 88' system was used for asymmetrical incorporation of engineered and native peptides (a 'type 8' system with more than 5–6 residues interferes with phage assembly); 5) a flexible linker was added to separate the SWNT-binding peptide from the fused coat protein.

Table S1: Sequences of the four peptides used in our study, called P4, P19, P23, and P28.

P4	H	N	W	Y	H	W	W	M	P	H	N	T
P19	S	S	A	W	W	S	Y	W	P	P	V	A
P23	H	S	S	Y	W	Y	A	F	N	N	K	T
P28	D	M	P	R	T	T	M	S	P	P	P	R

Table S2: Annealed duplexes prepared for cloning. *Sfi*I sites are colored in red, GGGS linkers in green, and SWNT-binding peptide inserts in black.

Inserts	Annealed duplexes
N4	5' TGGCCATAACTGGTATCATTGGTGGATGCCGCATAACACCGGCGGCGGCTCGGCCTCTG 3'<br>3' TGCACCGGTATTGACCATAGTAACCACCTACGGCGTATTGTGGCCGCCGCCGAGCCGGA 5'
N19	5' TGGCCAGAGCAGCGCGTGGTGGAGCTATTGGCCGCCGGTGGCGGGCGGCGGCTCGGCCTCTG 3'<br>3' TGCACCGGTCTCGTCGCGCACCACCTCGATAACCGGCGGCCACCGCCCGCCGCCGAGCCGGA 5'
N23	5' TGGCCATAGCAGCTATTGGTATGCGTTTAACAACAAAACCGGCGGCGGCTCGGCCTCTG 3'<br>3' T GCACCGGTATCGTCGATAACCATACGCAAATTGTTGTTTTGGCCGCCGCCGAGCCGGA 5'
N28	5' TGGCCAGGATATGCCGCGTACCACCATGAGCCCGCCGCCGCGTGGCGGCGGCTCGGCCTCTG 3'<br>3' TGCACCGGTCCTATACGGCGCATGGTGGTACTCGGGCGGCGGCGCACCGCCGCCGAGCCGGA 5'

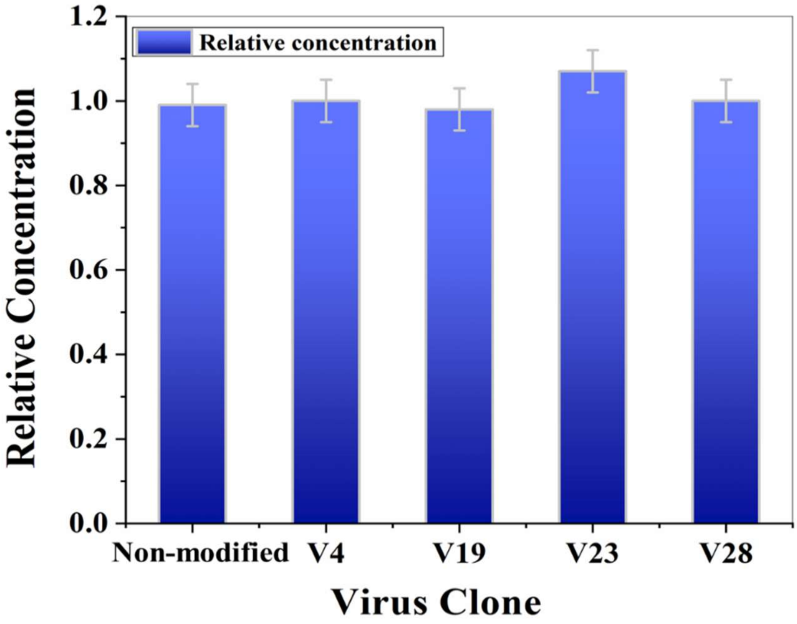

Figure S1: Diluted virus samples' quantification. Total virus proteins' relative concentration for each virus sample. Dilution indicates nearly equal overall phage levels for all five phage types (V4, V19, V23, V28, and native). These results indicate that all five phage types are present in equal amounts with similar relative concentrations of all virus samples.

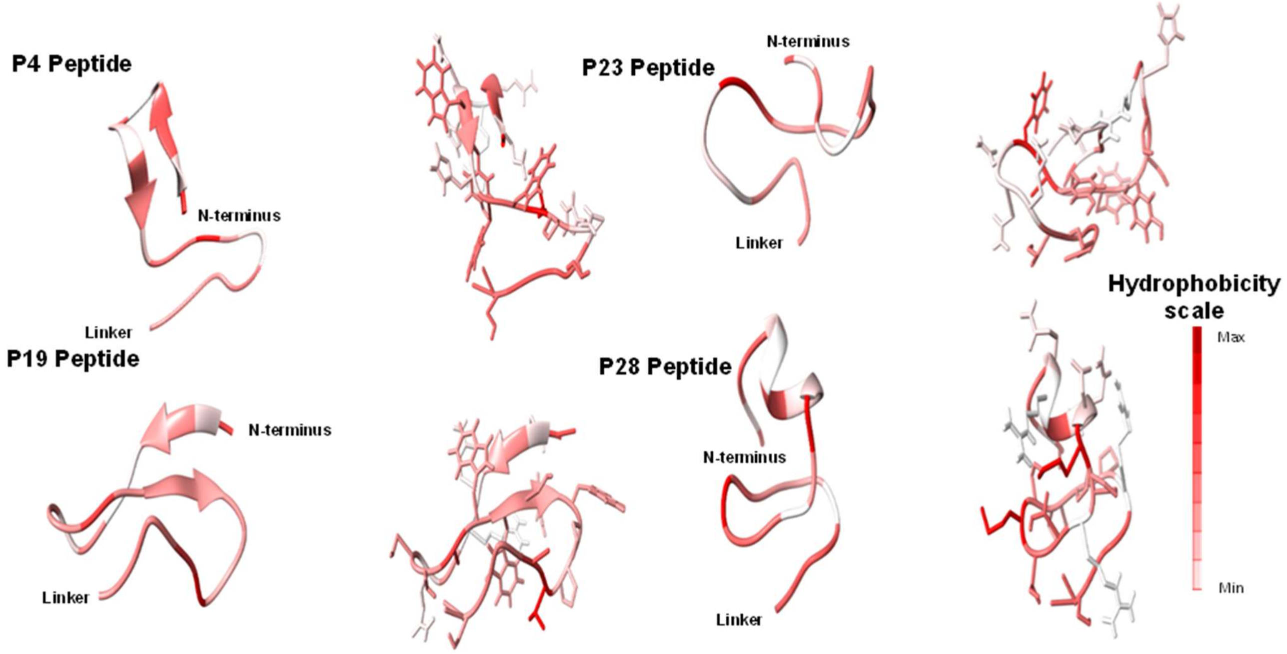

Figure S2: 3D ribbon models of P4, P19, P23, and P28 SWNT-binding peptides. Each peptide N-terminal end exposed to the solution and C-terminal linker ends are indicated. Right and left sides depict each peptide backbone with and without side chain residues, respectively. Colors indicate amino acid hydrophobicity; the red to white gradient indicates the maximal to minimal hydropathy, respectively.

3D theoretical ribbon models of the P4, P19, P23, and P28 peptides generated by the PEP-FOLD server; the online server is based on a hidden Markov model-derived structural alphabet (SA).
